# Characterization of a Moderately Virulent Pigeon Paramyxovirus Type 1 (Subgenotype VI.2.1.1.2.2) Strain: Genetic Evolution and Pathogenicity in Pigeons

**DOI:** 10.3390/v17111450

**Published:** 2025-10-31

**Authors:** Wuchao Zhang, Jiawei Chen, Hongze Pang, Baishi Lei, Kuan Zhao, Yunhang Zhang, Yinli Bao, Wenming Jiang, Wanzhe Yuan

**Affiliations:** 1 College of Veterinary Medicine, Hebei Engineering and Technology Research Center of Veterinary Biotechnology, Hebei Agricultural University, Baoding 071001, China; zhangwc@hebau.edu.cn (W.Z.); 15530215644@163.com (J.C.); hongzepang@163.com (H.P.); leibaishi2000@163.com (B.L.); zhaokuan@hebau.edu.cn (K.Z.); zhangyunhang@hebau.edu.cn (Y.Z.); 2 National Research Center of Engineering and Technology for Veterinary Biologicals, Nanjing 210014, China; 3 Key Laboratory of Fujian Universities Preventive Veterinary Medicine and Biotechnology, Longyan University, Longyan 364012, China; 82019007@lyun.edu.cn; 4 China Animal Health and Epidemiology Center, Qingdao 266000, China; jiangwenming@cahec.cn

**Keywords:** pigeon, paramyxovirus type 1, genetic evolution, pathogenicity

## Abstract

Pigeon paramyxovirus type 1 (PPMV-1) poses a significant threat to pigeon farming in China, and understanding its biological characteristics and pathogenicity is critical for vaccine development and disease control. In this study, we characterized a PPMV-1 QY strain, performed full-length genome sequencing, and constructed a phylogenetic tree based on the F gene. Then, the biological properties and the pathogenicity of the QY strain were assessed and evaluated in vitro and in vivo. The results showed that phylogenetic analysis classified the QY strain within subgenotype VI.2.1.1.2.2, the predominant circulating strain in China. The QY strain exhibited a 50% egg infectious dose (EID_50_) of 10^−6.8^/0.1 mL, mean death time (MDT) in chicken embryos of 68.7 ± 2.1 h, and intracerebral pathogenicity index (ICPI) in one-day-old chicks of 1.12, which indicate it is a moderately virulent strain. Animal experiments showed that the QY strain resulted in a mortality rate of 66.7% in healthy pigeons. Necropsy findings included cerebral congestion and swelling, hemorrhagic glandular stomach papillae, tracheal ring hemorrhages, and duodenal congestion and swelling. Histopathological analysis revealed extensive inflammatory infiltration in the lungs and liver, widespread intestinal erosion, and severe necrosis of splenic red pulp cells. In conclusion, the QY strain belongs to subgenotype VI.2.1.1.2.2 and exhibits moderate virulence, causing high mortality and severe pathological lesions in infected pigeons. These findings provide valuable insights into the pathogenicity of PPMV-1 and the specific mutations in the F protein can serve as potential attenuation targets in vaccine development against the emerging subgenotype VI.2.1.1.2.2.

## 1. Introduction

Pigeon plague, also known as pigeon Newcastle disease, is an acute, highly contagious infectious disease caused by pigeon paramyxovirus type 1 (PPMV-1), a variant of Newcastle disease virus (NDV) that primarily infects pigeons. Taxonomically, PPMV-1 belongs to the order *Mononegavirales*, family *Paramyxoviridae*, subfamily *Avulavirinae*, and genus *Orthoavulavirus* [1,2]. Although NDV exhibits a single serotype, it is genetically diverse, with over 20 recognized genotypes. PPMV-1 is classified within genotype VI, one of the prevalent NDV genotypes [3]. Virulence classification of PPMV-1 strains follows World Organisation for Animal Health (WOAH) criteria, which evaluate mean death time (MDT) in chicken embryos, intracerebral pathogenicity index (ICPI) in one-day-old chicks, and intravenous pathogenicity index (IVPI) in six-week-old chickens. Based on these parameters, PPMV-1 strains are categorized as highly virulent, moderately virulent, or low virulent [4,5].

PPMV-1 was first introduced into mainland China through Hong Kong in 1985 and subsequently spread across the eastern and southern regions. The epidemic exhibited incidence rates of 30–70% and mortality rates ranging from 40% to 80%, causing substantial economic losses to the pigeon industry [6,7]. Molecular epidemiological studies have identified subgenotypes VI.2.1.1.2.1 and VI.2.1.1.2.2 as the currently circulating dominant strains in China [8]. The virus demonstrates broad tissue tropism, primarily targeting the nervous, respiratory, and digestive systems. Infected pigeons present with a range of clinical manifestations, including neurological signs (lethargy, tremors, torticollis, paralysis, and ataxia), respiratory symptoms (tracheal hemorrhage and eyelid edema), and digestive disturbances (yellow-green diarrhea, intestinal hemorrhage, and hemorrhagic glandular stomach papillae) [9].

The present study was designed to investigate the biological characteristics and pathogenic potential of the PPMV-1 QY strain. Phylogenetic analysis was performed based on the complete F gene sequence. The biological properties of the QY strain were systematically evaluated through determination of EID_50_, MDT, and ICPI. Furthermore, pathogenicity assessment was conducted via pigeon challenge experiments. These findings clarify the genetic evolution and pathogenicity of the PPMV-1 QY strain, which not only provides essential data for optimizing diagnostic methods but also enables improved epidemic trend forecasting.

## 2. Materials and Methods

Virus Strain, Reagents and Animals. The PPMV-1 QY strain was sequenced and submitted to NCBI GenBank (Accession No. ON939087). Specific pathogen-free (SPF) chicken embryos were obtained from Jinan Sais Poultry Co., Ltd. (Jinan, Shandong, China). Experimental pigeons were confirmed with serum HI antibody titers against PPMV-1 below to 2^2^. The viral RNA extraction kit was purchased from Beijing TransGen Biotech (Beijing, China). The reverse transcription kit and PrimeSTAR^®^ Max DNA Polymerase were obtained from TaKaRa Biomedical Technology (Beijing) Co., Ltd. (Beijing, China). Agarose Regular was purchased from Shanghai Sangon Biotech (Shanghai, China). The agarose gel purification kit was acquired from OMEGA. The 2 × Taq Marker Mix and Gold View nucleic acid dye was purchased from ComWin Biotech (Taizhou, Jiangsu, China).

Phylogenetic Analysis. Full-length F gene sequences of reference NDV strains were retrieved from the NCBI database. Sequence alignment and comparative analysis were performed using MegAlign software (version 5.0) (Lasergene package, DNASTAR Inc., Madison, WI, USA). A phylogenetic tree was conducted based on the complete F gene coding sequence (1–1662 nt) using the neighbor-joining (NJ) method in MEGA 11, with 1000 bootstrap replicates.

Virus Titration. The viral solution was serially diluted 10-fold in sterile PBS to obtain dilutions from 10^−1^ to 10^−9^. Each dilution (0.1 mL per embryo) was inoculated into five SPF chicken embryos via the allantoic cavity. Embryos dying within 24 h post-inoculation were excluded as non-specific deaths. Allantoic fluid from the surviving embryos was harvested at 120 h post-inoculation for hemagglutination (HA) assay. The EID_50_ was calculated based on three independent replicates using the Reed–Muench method.

MDT Assay. Following 10-fold serial dilution in sterile PBS, the viral inoculum (0.1 mL/embryo) was administered to five SPF chicken embryos per dilution. Embryos were monitored for mortality at 37 °C over 7 days, with time of death recorded for MDT calculation.

ICPI Determination. Ten 1-day-old SPF chicks were inoculated intracerebrally with 0.05 mL of 1:10 diluted viral solution. Clinical symptoms were monitored and scored daily for 8 days (0 = normal, 1 = sick, 2 = dead). The ICPI was then calculated based on these scores.

Animal Experiment. Thirty 4-week-old healthy pigeons were purchased from a commercial pigeon farm (Baoding, China) and confirmed to be free of PPMV-1 antibodies. They were randomly divided into two groups (*n* = 15/group), an infection group and a control group. All pigeons were maintained under negative pressure isolators with free access to food and water. The infection group received an intramuscular injection (0.2 mL per pigeon) of viral allantoic fluid containing 10^6^ EID_50_ of PPMV-1, which was based on preliminary infection experiments and consistent with the doses commonly used in published PPMV-1 pathogenicity studies to mimic a natural infection, while the control group was injected with 0.2 mL of PBS [9]. Viral shedding was monitored via oropharyngeal and cloacal swabs collected at 1, 3, 5, 7, 10, and 14 days post-infection (dpi). Clinical signs and mortality were recorded throughout the experiment. Surviving pigeons were humanely euthanized at 14 dpi by an overdose of inhaled isoflurane followed by intravenous sodium administration. Necropsy was then performed, and the tissue samples were collected for histopathological analysis.

Statistical analysis. Statistical analyses were performed using one-way analysis of variance (ANOVA) test or two-way ANOVA test in the GraphPad Prism (version 6). *p* values of <0.05 were considered statistically significant; *p* values of <0.001 were considered extremely significant.

## 3. Results

### 3.1. Genetic Evolution Analysis

**Figure 1:**
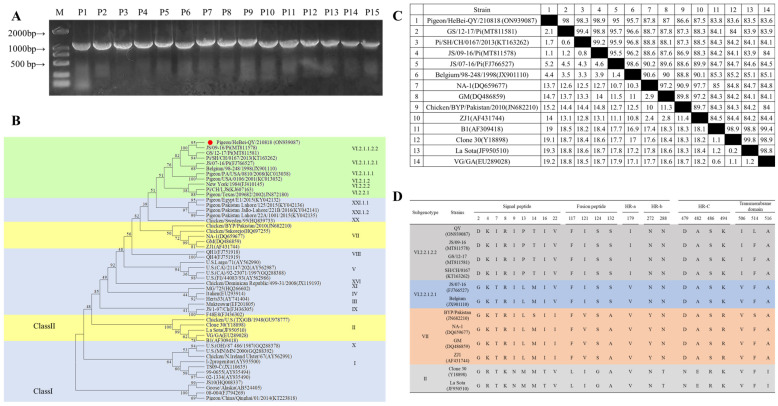
Genetic evolutionary analysis of the PPMV-1 QY strain. (**A**) PCR amplification of QY strain full-length genome. M: DL2000 DNA Marker; P1–P15: The PCR products of genome sequence of QY strain from 1–15. (**B**) Phylogenetic analysis based on PPMV-1 F gene nucleotide sequences. The red dot represents the virus strain used in this study. (**C**) Analysis of F gene nucleotide homology. The nucleotide homology is shown in the upper right corner, and the percent divergence is shown in the lower left corner. (**D**) Analysis of F gene amino acid differences.

The full-length genome of the QY strain was successfully amplified by PCR, yielding 15 products of the expected size (Figure 1A). Phylogenetic analysis based on the F gene sequence classified the QY strain within subgenotype VI.2.1.1.2.2, which represents the currently dominant lineage circulating in China (Figure 1B). Comparative sequence analysis of 14 representative F genes from subgenotypes VI.2.1.1.2.2, VI.2.1.1.2.1, as well as genotypes VII and II revealed that the QY strain shared 86.6–87.8% nucleotide identity with genotype VII strains, whereas it shared only 83.5–83.8% identity with genotype II vaccine strains (La Sota, B1, and Clone 30), demonstrating substantial genetic divergence between the QY strain and both genotype VII and II strains (Figure 1C).

Amino acid sequence comparison of the F protein identified key variation sites among the different genotypes. Compared to genotype II strains, subgenotype VI.2.1.1.2.2 strains exhibited 8 amino acid substitutions in the signal peptide region, 4 aa in the fusion peptide region, 6 aa in the heptad repeat regions (HR-a, HR-b, and HR-c), and 2 aa in the transmembrane region. When compared with genotype VII strains, these differences reduced to 5 aa in the signal peptide, 3 aa in the fusion peptide, 3 aa in the heptad repeat regions, and 1 aa in the transmembrane region. Notably, the QY strain displayed a unique single amino acid variation in the transmembrane region when compared with the other three VI.2.1.1.2.2 sequences. The cleavage site, being highly conserved, was not included (Figure 1D).

### 3.2. Biological Characteristics of the QY Strain In Vitro

**Figure 2:**
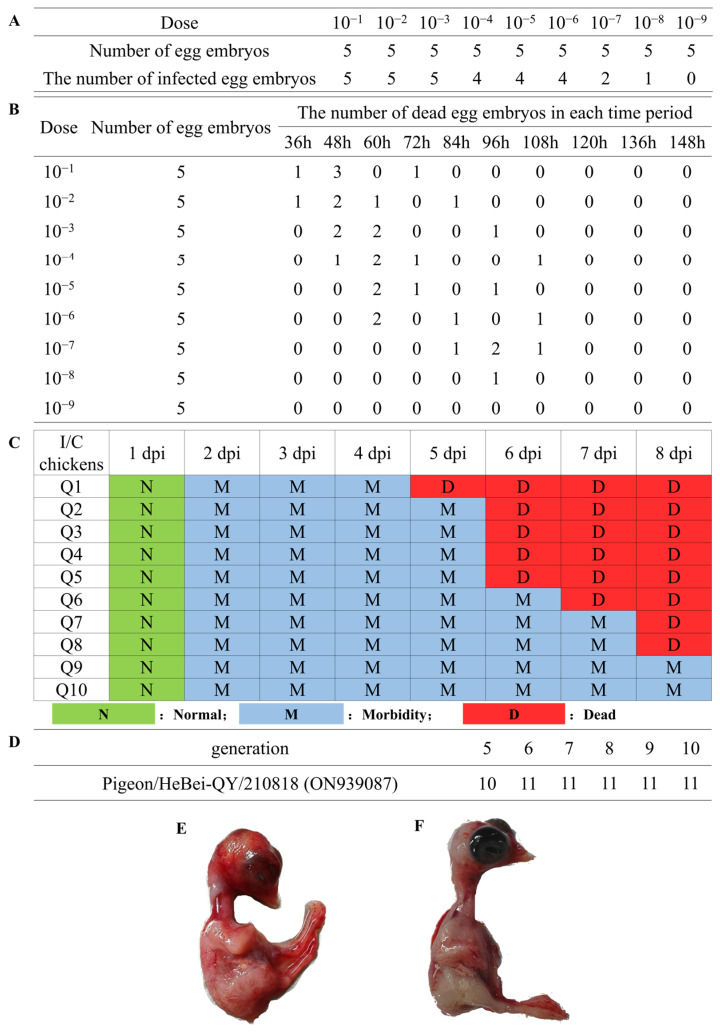
Biological characteristics of the PPMV-1 QY strain. (**A**) Determination of the 50% egg infectious dose (EID_50_). (**B**) Determination of the Mean Death Time (MDT). (**C**) Determination of the Intravenous Chicken Pathogenicity Index (ICPI). (**D**) Determination of hemagglutination (HA) titer. (**E**) Chicken embryos after QY strain infection. (**F**) Chicken embryos in control group.

The biological properties of the QY strain were assessed in accordance with WOAH standards for NDV virulence evaluation. Viral titration revealed an EID_50_ of 10^6.8^/0.1 mL (Figure 2A). Subsequent analysis demonstrated an MDT of 68.7 ± 2.1 h (Figure 2B) and an ICPI of 1.12 (Figure 2C), respectively. Serial passage experiments in SPF chicken embryos showed that the QY strain exhibited a consistent HA titer of 1:2^11^ across passages 5 through 10 (Figure 2D), indicating stable viral replication kinetics. In addition, pathological examination revealed extensive hemorrhagic lesions throughout the infected embryos, presenting as diffuse red spotting in contrast to normal controls (Figure 2E). Collectively, these results classified the QY strain as a moderately virulent strain.

### 3.3. Pathogenicity Analysis of the QY Strain in Pigeons

**Figure 3:**
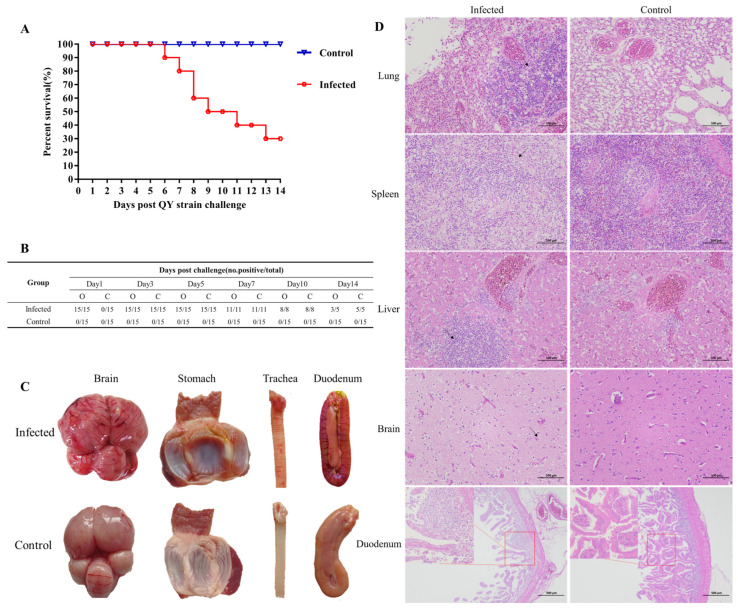
Pathogenicity analysis of the PPMV-1 QY strain. (**A**) Survival rate of pigeons in QY strain-infected and control groups. (**B**) Viral shedding detection in pigeons inoculated with QY strain. O: Pharyngeal swab; C: Cloacal swab. (**C**) Postmortem examination of pigeons in QY strain-infected and control groups. (**D**) Histopathological examination of experimental pigeons. Lung, Spleen, Liver, Brain: 200×; Doudenum: 40×; The arrow denotes the lesion, and the red box delineates the area of local magnification.

The pathogenicity of the QY strain was evaluated through experimental infection of pigeons. The infected group exhibited a mortality rate of 66.7% (10/15), with initial deaths occurring at 6 days post-infection (dpi) and cumulative mortality reaching 10 birds by 14 dpi. This mortality rate was consistent with a previous report following intra-oculonasal infection [4]. No mortality was observed in the control group throughout the study period (Figure 3A). Viral shedding dynamics were monitored through RT-PCR analysis of oropharyngeal and cloacal swabs (Figure 3B). All infected pigeons demonstrated oropharyngeal virus shedding beginning at 1 dpi, with persistent detection through 10 dpi, and 60% positive rate at 14 dpi. Cloacal shedding was first detected at 3 dpi and continued through the study endpoint. The detoxification time occurred one day earlier than that reported by Chang et al. in pigeons infected via the intraoral route [9]. In contrast, no viral shedding was detected in control animals at any time point. Gross pathological examination of infected pigeons revealed characteristic lesions, including cerebral congestion, hemorrhage and swelling, hemorrhagic glandular stomach papillae, ring-shaped hemorrhages in the trachea, and congestion with swelling in the duodenum. In contrast, these pathological findings were absent in the control group pigeons (Figure 3C).

Histopathological examination revealed significant tissue damage in QY strain-infected pigeons compared to control pigeons (Figure 3D). The lungs in QY strain-challenged pigeons exhibited a large amount of inflammatory cell infiltration, cell necrotic debris, and obscured pulmonary architecture with indistinct capillary structures. The spleen displayed widespread necrosis of red pulp cells (indicated by arrows), nuclear dissolution, and cytoplasmic disintegration, along with eosinophilic flocculent deposition. For the liver, extensive lymphocyte infiltration could be seen around the liver parenchyma and the portal area (indicated by arrows), with a mild hepatocyte edema and cytoplasmic vacuolization. The brain presented multiple symptoms, including cerebral vascular congestion (indicated by arrows), and a large amount of lymphocyte infiltration, degeneration and necrosis of neurocytes. Additionally, the intestinal tissue showed extensive mucosal erosion, loss of normal villous architecture, and intestinal villi necrosis and abscission, accompanied by a small amount of lymphocyte infiltration (Figure 3D). Conversely, tissues from the control pigeons had minimal or no lesions and maintained normal histological architecture (Figure 3D). Minimal inflammatory cell infiltration in the lungs, with clear pulmonary and respiratory capillary structures. The spleen preserved normal red pulp with intact cell structures. The liver showed mild cell infiltration, with normal hepatocyte morphology and structure. The brain had abundant neurons with no significant inflammatory cell infiltration. The intestinal tissue had well-structured villi, with no obvious abnormalities (Figure 3D).

## 4. Discussion and Conclusions

PPMV-1 represents a pigeon-adapted variant of Newcastle disease virus (NDV), classified within genotype VI of Class II NDV. While these genotype VI strains exhibit primary tropism for pigeons, the genotype of these viruses demonstrates zoonotic potential, with documented infections in various avian species, including chickens, ducks, geese, turkeys, and quails, albeit typically with attenuated pathogenicity [10,11]. Of particular concern is the demonstrated capacity of low-pathogenicity genotype VI strains to regain virulence following serial passage in chickens, posing a potential biosecurity threat to the poultry industry [12]. Since PPMV-1 was introduced into China in 1985, the virus rapidly disseminated across regions, causing significant economic losses to the pigeon farming industry. Prior to 2010, the prevalent PPMV-1 in China belonged mainly to subgenotypes VI.1 (formerly VIb) and VI.2.2.2 (formerly VIe). At present, the dominant strains are subgenotypes VI.2.1.1.2.1 (formerly VIj) and VI.2.1.1.2.2 (formerly VIk), with the latter exhibiting increasing predominance [13]. Comparative genomic analysis reveals significant nucleotide and amino acid sequence divergence between the QY strain and representative genotype VII and II strains, particularly in key functional domains of the F protein. These genetic variations likely contribute to observed differences in host specificity and virulence patterns, providing critical targets for future research on PPMV-1 host adaptation and pathogenicity mechanisms.

According to the WOAH classification standards for NDV virulence, the QY strain demonstrated moderate virulence characteristics, with an MDT of 68.7 ± 2.1 h and ICPI of 1.12. These metrics position the QY strain within the moderate virulence category among circulating PPMV-1 isolates. The virulence and pathogenicity of the virus is primarily determined by the F protein. Specific amino acid mutations at critical sites are likely a key determinant of its virulence, a hypothesis that warrants further investigation.

Epidemiological investigations have established that the majority of PPMV-1 strains belonging to subgenotype VI.2.1.1.2.2 exhibited high pathogenicity in pigeons. Clinical observations of infected pigeons consistently reveal a constellation of severe symptoms, including pronounced lethargy, characteristic greenish diarrhea, wing ptosis, progressive paralysis, and terminal prostration, frequently culminating in fatal outcomes. A representative study by Tong et al., employing the QH-01 strain (VI.2.1.1.2.2), documented remarkably rapid disease progression, with initial mortality events occurring as early as 3 days post-infection (dpi) and ultimately reaching 80% case fatality in experimental period [14].

In the present study, pigeons challenged with the QY strain exhibited comparable clinical manifestations, with disease onset typically manifesting at 4 dpi through neurological signs (torticollis), systemic symptoms (lethargy), hypersalivation, and distinctive yellow-green diarrheal discharge. Necropsy analyses identified consistent hemorrhagic lesions across multiple organ systems, particularly affecting the brain, trachea, glandular stomach, and duodenum. Histopathological evaluation further demonstrated extensive inflammatory cell infiltration and necrotic changes in parenchymal tissues, although the study was limited by the absence of immunohistochemistry (IHC) analysis. The infected group exhibited a mortality rate of 66.7% (10/15), with initial deaths occurring at 6 dpi and cumulative mortality reaching 10 birds by 14 dpi. The above data confirms the QY strain maintains the characteristically high virulence phenotype associated with this subgenotype. In addition, as for epidemiological significance, comprehensive virological monitoring revealed persistent viral excretion in both oropharyngeal (1–14 dpi) and cloacal (3–14 dpi) secretions across all challenged pigeons. This prolonged shedding profile, particularly notable from 3–14 dpi, presents substantial risks for environmental contamination and fomite transmission. Considering the exceptional mobility of urban and racing pigeon populations, such extended shedding periods dramatically amplify the potential for widespread viral dissemination across geographic regions. Hence, when evaluating vaccines against PPMV-1, in addition to assessing the kinetics and titers of serum-neutralizing antibodies, key efficacy criteria should include the reduction in viral shedding rate and the shortening of shedding duration. These virological parameters are critical indicators of a vaccine’s ability to effectively prevent PPMV-1 infection and transmission.

Vaccination remains the primary strategy for PPMV-1 prevention in China, yet significant challenges persist with existing immunization approaches. The commercially available vaccines, including the La Sota, Clone 30, and B1 strains, were originally developed for NDV in chickens and exhibited substantial genetic and antigenic divergence from PPMV-1 strains. Notably, PPMV-1 belongs to genotype VI, while prevalent NDV field strains in poultry are predominantly genotype VII [15,16]. Our sequencing analysis revealed only an 83.5% nucleotide homology in the F gene between the QY strain and La Sota, further highlighting this mismatch. Consistent with this genetic disparity, Qiu et al. demonstrated that La Sota-vaccinated pigeons developed HI titers of merely 1:102–1:94 against PPMV-1, whereas a homologous PPMV-1 vaccine elicited significantly higher titers (1:256). These findings underscore the limited cross-protection conferred by conventional NDV vaccines against PPMV-1. Similarly, Hamouda reported that the La Sota vaccine conferred only 60% protection in pigeons challenged with a virulent PPMV-1 strain, whereas a homologous PPMV-1-based vaccine achieved complete protection (100%) and effectively suppressed viral shedding. These results unequivocally demonstrated the limited efficacy of heterologous NDV vaccines against PPMV-1 and emphasized the critical need for vaccines specifically tailored to prevalent PPMV-1 genotypes. The comprehensive biological and pathogenic characterization of the QY strain presented in this study establishes a foundation for developing safe and efficacious PPMV-1 vaccine candidates. Based on the genetic profile of the QY strain, future work could focus on (i) evaluating the cross-neutralization efficacy of current La Sota-based vaccines against the subgenotype VI.2.1.1.2.2, and (ii) developing a reverse genetics system for the QY strain to generate a chimeric vaccine by inserting its F and HN genes into the attenuated La Sota backbone.”

In summary, this study genetically and pathogenically characterizes a pigeon-derived paramyxovirus type 1 (PPMV-1) strain QY. Phylogenetic analysis classifies it into subgenotype VI.2.1.1.2.2 and identifies key molecular variations distinguishing it from genotypes II and VII. Biological assessment reveals moderate virulence, broad tissue tropism, and sustained viral shedding, contributing to understanding PPMV-1 evolution and pathogenicity. These findings offer valuable insights for epidemiological surveillance and foundational data for future vaccine development.

## Data Availability

The original contributions presented in this study are included in the article. Further inquiries can be directed to the corresponding author.
